# Servant Leadership in a Social Religious Organization: An Analysis of Work Engagement, Authenticity, and Spirituality at Work

**DOI:** 10.3390/ijerph17228542

**Published:** 2020-11-18

**Authors:** Mar Ortiz-Gómez, Antonio Ariza-Montes, Horacio Molina-Sánchez

**Affiliations:** 1 Financial Economics and Accounting Department, Universidad Loyola Andalucía, 14004 Córdoba, Spain; hmolina@uloyola.es; 2 Social Matters Research Group, Universidad Loyola Andalucía, 14004 Córdoba, Spain; ariza@uloyola.es

**Keywords:** authenticity, work engagement, spirituality at work, corporate governance, servant leadership, religious organizations, third sector

## Abstract

Religious organizations represent a main part of the third sector and the social economy. Social faith-based institutions have some unique features that, in some respects, differentiate them from other entities, as they are characterized and defined not only by the services they provide, but also by how they provide them. It is part of their mission to convey the values that prevail in their institutional culture while developing their activities, being attractive to those workers who identify with their values. From this point of view, a key element of these entities’ success is that their employees feel identified with their work so that they are engaged in the institution and its values. The style of leadership exercised in such organizations is critical to fostering these attitudes and their long-term survival. This paper aims to study the link between perceived servant leadership by followers and work engagement, as well as the mediating role of authenticity and spirituality at work in this relationship. To this end, 270 workers from a Spanish Catholic organization in the social sector were surveyed. These data were processed by PLS (partial least squares). The results show that a servant leadership style by itself does not directly promote work engagement among employees of the target organization. The engagement of these workers comes through two mediating variables: authenticity and spirituality at work. This study covers a gap in the literature because although there are studies arguing that a strategy of servant leadership is critical to these organizations, to our knowledge, they do not finish demonstrating the fundamental roles that attitudes of authenticity and spirituality at work play in the perception of this type of leadership, achieving greater work engagement.

## 1. Introduction

Religious organizations now represent essential players in the third sector and the social economy in areas such as exclusion, disease, and education. Particularly, social religious organizations are typically a relevant part of any country’s service sector. The purpose of these entities does not only lie in the services they carry out, but also in how they provide their activities, which transmits their character and charisma. Conveying the values that prevail in their institutional culture is part of their mission [1]. Therefore, for social religious organizations, it is necessary to define specific organizational objectives that enable the achievement of their institutional mission while distinguishing them from other entities.

All of the above considerations suggest that workers are a critical component of these institutions because if employees share the values of the organization, they will help these institutions fulfill their mission of transmitting specific values while providing a service. Similarly, workers are key to ensuring the quality of services provided by these organizations and, therefore, to achieving long-term sustainability and viability. Religious organizations are currently facing significant challenges, that require them to set themselves apart from other entities working in their field of activity as well as rapid and important changes in the ways of life of society. All this, together with a context of promoting greater collaboration with the laity [2,3], make it crucial to the success of these entities that their employees feel comfortable and identify with the institution and their values so that, in this way, they show engagement in their work [1].

In this context, the style of leadership exercised in these organizations is critical to their long-term survival, and this is one of the research topics most studied for its influence on behavior [4]. Due to the importance of these entities carrying out their social and spiritual mission, this paper considers as a starting point that servant leadership is one of the leadership styles most consistent with social religious entities since it implies an approach based on moral values and ethical principles [5,6], as well as on religious teachings [7] It is related to Judeo-Christian philosophical traditions [8]. Servant leadership is a management strategy that prioritizes and turns workers’ needs into objectives, putting employees’ good above the leader’s self-interest and showing concern for others [9,10,11]. Although followers are the main focus of servant leaders, most attention in leadership theory is on leaders instead of followers. However, the perception of the employees is what is going to determine their attitudes. Different studies have shown that this type of leadership has many advantages on followers, such as increasing work engagement [12,13], authenticity [14], and worker wellbeing [10,11].

A positive association exists between work and personal life in many environments. The benefits of work engagement are not limited to the workplace. It also improves the quality of personal life and what healthcare calls good social functioning, such as improving family relationships [15,16]. Employees often search for the meaning of work and how their jobs meet with their lives [17]. The fact that religious organizations prioritize social objectives makes many of their employees value their jobs because they feel identified with the mission of the institution and with the impact this entity has in carrying out its activities [1,18]. For this reason, this paper explores the relevance of spirituality at work and authenticity for servant leadership and work engagement from the followers’ perspective. There is a growing need for empirical research on authenticity in the workplace [19], and it is important to investigate the wide range of positive results promoted by spirituality at work [20].

Therefore, given these unique characteristics of faith-based entities in the social sector, it is essential to understand how their members feel and act to achieve the organizations’ long-term sustainability and viability. While some previous research has already provided valuable information to understand how individual links work among perceived servant leadership, spirituality at work, authenticity, and work engagement, research on religious organizations is virtually non-existent, emphasizing the importance of this study. To this end, this article seeks to study the extent to which the style of servant leadership perceived by workers generates greater work engagement, as well as the influence on this relationship of individual attitudes characterized by spirituality at work and authenticity among workers of a social religious organization.

A faith-based institution in the social sector is an appropriate context to study previous links because these workers generally differ from those in other types of organizations by a compendium of two characteristics. First, it is a social organization, and second, it is religious. On the one hand, the social component reflects that it is mainly a vocational work, due to the intense demands that many of the social jobs require, such as dealing with battered women or children with severe disabilities or other situations of social exclusion. On the other hand, the religious component determines that the organization has a particular mission, vision, and values. This fact makes that the organization does not care only for the provision of social services, but also that their personnel transmit the values of their vision. They look for motivated professionals identified with the mission and values, that help them to develop their activity according to the organizational culture. Both circumstances, social and religious, make that these workers value a servant leadership strategy, as probably it shares many of the characteristics of their personality and personal values; their work engagement is likely to be greater than in other sectors of activities where they may do not demand a vocation, or they do not share the values of the vision of the institution. Hence, due to all the circumstances explained above, it is likely that they have a greater attitude of authenticity and spirituality at work.

Moreover, these relationships are of great importance also for general knowledge, as there are also other nonprofit and profit organizations that follow a style of servant leadership, due to their mission and strategy, or due to the wide range of generated outputs commented above, such as work engagement. Attitudes of authenticity and spirituality at work in followers probably affect how a strategy of servant leadership is perceived by the workers of any type of organization, and then, their engagement to their jobs. If employees are free to live in accordance with their spirituality and their values and beliefs, they are going to feel more comfortable and engaged in their works. To our knowledge, the influence of these individual attitudes has not been studied yet in an organizational environment. Furthermore, most of the research analyzes servant leadership from the leader´s perspective. Attitudes that followers show in their work are going to depend on how they perceive their leaders, rather than how leaders perceive their own attitude and behavior towards their employees.

The rest of the article is structured as follows. The next section sets out the theoretical framework and research hypotheses. The methodology section details the methods used to meet the objectives of the research. The results obtained are presented below. The discussion section provides the most relevant empirical results. Finally, the article summarizes the main conclusions, as well as the main implications and limitations.

## 2. Theoretical Framework and Research Hypotheses

### 2.1. Servant Leadership and Work Engagement

The concept of servant leadership was introduced by Greenleaf [21] five decades ago, who argues that servant leaders prioritize stakeholders’ interests over personal ones and, unlike in other leadership strategies, understand their position as a vehicle for serving workers, the organization, and the community. Greenleaf [21] and Spears [22] visualize this willingness to help and serve the progress of individuals and groups as committing to building a community in the workplace, listening receptively to others, developing a high level of empathy, and relying more on persuasion than coercion.

Servant leadership is based on the beliefs of promoting value and development in individuals, sharing power for the common good, building a community, and exercising authenticity [14]. From this point of view, Reinke [8] believes that a servant leader is dedicated to the growth of both the worker and the organization and tries to build a community within the organization. This author states that servant leadership is a relationship, not a position, that prioritizes the needs of others and the entity.

Reinke [8] defines servant leadership as a three-dimensional construct composed of openness, stewardship, and vision. To identify these three dimensions, this author draws from the ten key characteristics of servant leadership that Spears [6] defines. First, openness involves Spears’ elements of empathy, listening, and awareness of others. Second, stewardship includes four of Spears’ concepts that Reinke considers intimately intertwined: healing, commitment to the growth of individuals, persuasion, and stewardship. It concerns a participatory leadership style in which a servant leader prioritizes the needs of workers and the organization and is devoted to their development. Finally, vision dimension refers to the ability to contextualize circumstances and look at them in perspective to predict and plan for future necessities.

A wide variety of authors have found that the perception of servant leadership generates positive effects on workers’ engagement [12,13,23]. Work engagement refers to the positive and persistent emotional affective state of employees, which is characterized by absorption, dedication, and vigor [24]. Absorption refers to the state of concentration in which the employee is happily immersed in his work and time seems to pass quickly. Dedication leads to inspiration, participation, meaning, challenge, enthusiasm, and pride. Finally, vigor means dedication to work, with energy, pleasure, and effort, despite the difficulties that it may entail. The advantages of work engagement for the organization are innumerable, since those employees who are engaged may do a better job than those who are not [25].

This study focuses on behavioral theories to build the relationship between servant leadership and work engagement. These theories that defend that servant leadership transform their followers’ mindset, attitudes, and behaviors are in line with Greenleaf´s theory [21], in favor of servant leadership as a relationship and emphasis on the connection between the leader and followers. This author centers servant leadership on attitudes and explains that servant leaders are likely to create transforming effects on followers and remodel them into servant leaders themselves. In this line, behavioral theories of social learning and social identity could be useful to explain the aforementioned relationships. Social learning theory [26] defends that followers observe and then emulate the attitudes, values, and behaviors of the servant leader, as they are likely to be considered credible role models that act altruistically and are motivated to serve others, and hence, in last instance, they influence performance [10]. In this sense, as a servant leader is committed to selflessly serve their employees and the organization, when employees observe this behavior in a positive service climate, they are likely to feel motivated to develop these attitudes of commitment and increase their work engagement.

Social identity theory [27] explains that leaders could change or create specific employees´ behaviors, modifying their self-identity or part of the self-concept that determines their emotional attachment to the group. This theory has been used to explain that, due to the authentic and follower centric nature of servant leadership, leaders make followers feel equal in the organization by developing tight bonds with them. Once workers self-identify with the group, they are more likely to engage in beneficial behaviors for the organization [28]. For instance, some previous research has shown that servant leadership reduces burnout [29]. In this line, servant leaders promote employees’ self-identity with the group and create strong bonds with them, through their support and coaching [30], involving them in the planning and decision making [14], or listening receptively to them [21,22]. Servant leaders also promote the building of a community in the workplace. This sense of being part of a group and belonging to a community provides followers with meaning and identity [31]. Hence, followers who feel this identification with the group probably are more likely to become more involved and engaged in their work tasks. In the service sector, servant leadership may build this sense of social identity in their followers intensively, and ultimately their service performance, due to the people-centered, unpredictable, and dynamic nature of this industry [28].

Furthermore, the concept of servant leadership has its roots on religious teachings [7], and Christ is considered as a model for servant leaders among Judeo-Christian cultures [8]. Hence, values promoted by this leadership style probably coincide with the ones of religious entities and this supported link between servant leadership and work engagement should also work among workers in faith-based entities. Based on the previous arguments, Hypothesis 1 (H1) of this paper is as follows:

**Hypothesis** **1.** *Servant leadership is positively related to work engagement among workers in social religious organizations*.

### 2.2. The Mediating Role of Spirituality at Work

Spirituality at work does not refer only to the religious aspect [32]. This concept is based on the values and philosophy of each person [33]. Values allow individuals to find their life purpose at work, to feel a strong connection with the organization, and to enjoy an alignment of their values and beliefs with those of the organization. This paper is based on the concept of spirituality of Milliman et al. [33], who define spirituality at work as a construct with three dimensions, which were selected by these authors based on the seven dimensions identified by Ashmos and Duchon [34]. The first dimension, called meaningful work (individual level), not only refers to having a pleasant job or being energized by work but also implies that workers’ personal lives nurture and feed off meaningful work, thus contributing to finding meaning and personal purpose. The second dimension refers to having a sense of community (group level), which implies a deep connection between co-workers, including support and genuine care, as well as being linked by a common purpose. Finally, the third dimension, alignment with the organization´s values (organization level), involves sharing the values and mission of the organization. It means that employees feel connected to the entity’s goals, mission, and values. It is based on the belief that all members of the organization care about the employees’ and community’s wellbeing, as well as in the organization’s concern for employees.

Servant leadership is intrinsically related to spirituality at work, which is a great source of motivation for these leaders [35]. There are even authors such as Sendjaya et al. [36] who argue that servant leadership emphasizes a spiritual orientation and consider spirituality a *sine qua non* dimension of this type of leadership. Other researchers, such as Khan et al. [37], find that servant leadership has a positive and significant effect on spirituality at work, working with a sample of 214 employees in organizations at the governmental and private level of Pakistan. They explain that this relation comes from the fact that the concept of servant leadership has roots in religious teachings, so in the context of this research, a faith-based organization that tries to promote their religious values among their employees, this relation acquires importance. Hence, servant leadership could also be a valued tool to improve workplace spirituality in faith-based institutions.

Additionally, the behavioral leadership theories could be used to look for a possible explanation of the positive relationship between servant leadership and the three dimensions of spirituality at work. Following the social learning theory [26], servant leadership makes employees improve their perception of alignment with organizational values. If employees perceive their leaders as a credible role model that share the values of the organization, they are going to emulate these attitudes, values, and behaviors, and hence, they are going to feel connected to the entity’s values, goals, and mission. Following the social identity theory [27], the follower centric and developmental nature of servant leadership makes employees increase their perception of meaningful work [37], as servant leaders serve and care about their followers, prioritizing their interests, helping in their progress, listening to them, and sharing power [21], developing strong bonds with them. Servant leaders are also committed to creating a community in the organization, what probably makes employees self-identify with the group, and hence creates a sense of community. Hypothesis 2a (H2a), therefore, states the following:

**Hypothesis** **2a.** *Servant leadership is positively related to spirituality at work among workers in social religious organizations*.

Spiritual beliefs reinforce work engagement [38] because religious and spiritual perspectives can affect how people perceive the circumstances of their daily lives or how they structure their activities and their global sense of wellbeing and satisfaction with life [39]. In this vein, a longitudinal study of Christian religious workers (clerics, chaplains, missionaries from various cultures, and other employees within religious organizations) conducted in Australia showed that the religious bond generates greater work engagement among these people than in other groups of individuals, increasing the meaning of tasks and the perceived capacity to successfully perform them [40].

In line with the above and according to Milliman et al. [33], spirituality at work plays an important role in promoting work engagement. In organizations with strongly marked and socially oriented values, worker alignment with the organizations’ values should increase work engagement. Furthermore, employees who have a great sense of community and consider their work to have a purpose and are aligned with the organization’s values should also feel intensely engaged. Therefore, Hypothesis 2b (H2b) suggests the following:

**Hypothesis** **2b.** *Spirituality at work is positively related to work engagement among workers in social religious organizations*.

These last two hypotheses form Hypothesis 2 (H2), which asserts that spirituality at work is a mediating variable between servant leadership and work engagement:

**Hypothesis** **2.** *Spirituality at work mediates the relationship between servant leadership and work engagement among workers in social religious organizations*.

### 2.3. The Mediating Role of Authenticity at Work

Authenticity is mainly about acting according to one’s values and beliefs [41]. This research takes as its starting point the definition of authenticity by Rogers [42], which, focusing on the person, is an attitude that allows the full functioning of human beings. Following this idea, Wood et al.´s [43] multidimensional authenticity model is shaped around three fundamental dimensions: authentic living, self-alienation, and accepting external influence; achieving the optimum level of authenticity when authentic living reaches a high level, and self-alienation and acceptance of external influence present low levels. Authentic living refers to being true to oneself and behaving according to one’s own beliefs and values. Accepting external influence is meeting the expectations of others, in other words, to what degree an individual is influenced by other people’s thoughts and actions. Finally, self-alienation refers to a state in which an individual experiences inconsistency between an experience and who he/she is; self-alienation translates to the workplace as not knowing whom one is at work. Following the recommendations of Goldman and Kernis [44], this multidimensional model of authenticity is very suitable for research in the workplace. In the specific case of the target organization, it acquires a particular interest to go deeper in this concept, due to the range of demands that in many cases, employees of social entities have to face.

The academy has also linked servant leadership to the concept of authenticity [14]. Servant leadership applies the authentic attributes of authentic leadership. Van Dierendonck and Heeren [45] argue that a servant leader is characterized by authenticity, integrity, humility, courage, and objectivity. To be authentic refers to act according to one’s values and beliefs [46], and servant leaders experience their lives according to the values they have acquired [47]. In this line, based on social learning theory [26], if employees observe these explained attitudes of authenticity in their servant leaders, they would be likely to emulate the same ones. On the other hand, social identity theory [27] refers to how servant leaders make employees feel. In this sense, servant leaders try to achieve transparency in their workers and consistency between what they say and do [36]. Hence, as servant leadership creates a climate of trust [12] and authenticity [14] among their followers developing strong bonds, they are likely to feel more comfortable to be authentic in the workplace. Another characteristic of servant leaders that could determine the authenticity of their followers through the social identity theory is empowerment. A servant leadership culture empowers employees to grow freer, more independent and selfless, giving them freedom of decision making [12], which will probably generate in followers a higher perception of being able to act in accordance to their values and beliefs. Therefore, Hypothesis 3a (H3a) of this paper states the following:

**Hypothesis** **3a.** *Servant leadership is positively related to authenticity at work among workers in social religious organizations*.

In addition, the proposed research model seeks to test whether authenticity increases work engagement. Academics from a wide range of disciplines have attracted attention to the intensified search for authenticity in developed cultures [46], as it has a wide variety of positive effects on workers because it gives meaning to their work [48]. This issue is increasingly relevant, as being authentic benefits individuals and groups, which contributes to creating healthier organizations. When workers are forced to develop behaviors contrary to their values and beliefs, different types of psychopathologies are generated [41].

Studies such as those of Van den Bosch and Taris [49,50] conclude that the more authentic employees are in their work, the better they adapt to it and the more energetic they feel, becoming more engaged in the work. Van den Bosch and Taris [50] show that authenticity at work represents on average 11% of the variance of the result variables studied in the research, which include work engagement. Of the three dimensions of authenticity at work, these authors identify self-alienation as the strongest predictor of work engagement, followed by authentic living and accepting external influence. Using a sample of employees of a religious organization, Ortiz-Gómez et al. [1] confirm that those workers who feel that they can act according to their values and beliefs in their work environment are more engaged in their work. Based on this argument, Hypothesis 3b (H3b) states the following:

**Hypothesis** **3b.** *Authenticity at work is positively related to work engagement among workers in social religious organizations*.

These last two hypotheses form Hypothesis 3 (H3), which states that authenticity at work is a mediating variable between servant leadership and work engagement:

**Hypothesis** **3.** *Authenticity at work mediates the relationship between servant leadership and work engagement among workers in social religious organizations*.

Finally, Hypothesis 2, together with Hypothesis 3, make up Hypothesis 4 (H4), which proposes the following:

**Hypothesis** **4.** *Spirituality and authenticity at work mediate the relationship between servant leadership and work engagement among workers in social religious organizations*.

**Figure 1:**
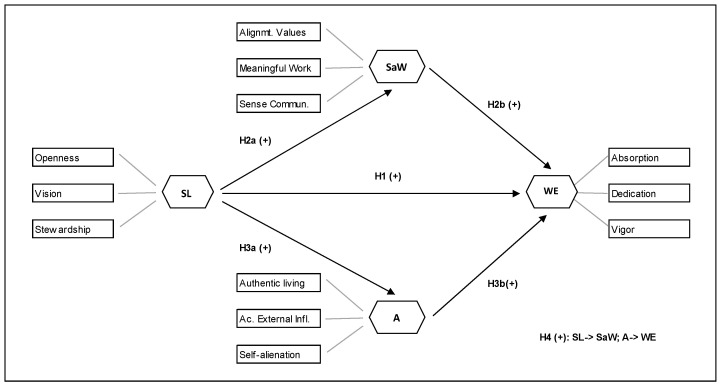
Research model and hypothesis; A, authenticity; SaW, spirituality at work; SL, servant leadership; WE, work engagement.

Figure 1 depicts both the research model and the previous assumptions.

## 3. Methodology

### 3.1. Participants and Data Collection

The objectives of this research were met through a self-administered questionnaire that was sent in July 2019 through Google Forms to all the workers of a Spanish religious organization that conducts activities in the social sector. The target entity is a statewide nonprofit Catholic organization whose mission, within the framework of the promotion and defense of human rights, is to carry out social intervention projects, helping to the integral development of people in a situation of risk or social exclusion. This religious organization that operates in the south of Spain has 30 social centers and undertakes different social intervention projects, such as day services, socio-labor insertion, support for immigrants, family intervention, and equal opportunities for women. Of a target sample of 499 workers, 283 responded to the survey, and, after a checking process eliminating questionnaires with missing values, the final sample consisted of 270 valid questionnaires (54.1%). Participation was voluntary and anonymous, and all the respondents provided signed informed consent for the study. All participants were informed about the content and the characteristics of the research before completing the questionnaire. It was carried out following the Helsinki Declaration and was approved by the Ethics Committee of Loyola University.

Of the 270 valid responses, 76% are from non-manager employees, and the remaining 24% are from respondents in manager positions. Women make up 71.5% of the respondents, and only 28.5% are men. Their average age is 38 years (SD: 8.1), and their average seniority in the organization is 4 years (SD: 4.9). Most of them work on the following projects: socio-labor insertion (39%), socio-educational (29%), and residential (29%); the remaining 3% work in central services, territorial and socio-labor management, employment, summer school, and youth justice. Most of the employees have completed high-level studies: 23% have a master’s or PhD, 68% have a university education, and the remaining 9% have finished secondary or primary studies.

### 3.2. Questionnaires and Scales of the Variables Analyzed

All the variables in this study were measured through validated questionnaires that have, therefore, previously demonstrated their reliability. Perceived servant leadership was measured by the Spanish version of Ortiz-Gómez et al. [5] SSLS6-3F (Spanish Short Servant Leadership Survey), which was developed from the original version of Reinke [8]. It evaluates the perception of servant leadership of the immediate supervisor of each worker. This Spanish scale of servant leadership contemplates the three dimensions identified in the theoretical framework, composed each of them by two items (openness, i.e., “my supervisor listens to what employees have to say”; stewardship, i.e., “my supervisor is committed to helping employees grow and progress”; and vision, i.e., “my supervisor emphasizes doing the right thing for the long-term benefit of all”), evaluated by a Likert scale ranging from 1 (low perception of servant leadership in his/her superior) to 5 (high perception of servant leadership). The estimated reliability of the three subscales was 0.83 for openness, 0.87 for vision, and 0.83 for stewardship [5].

To assess work engagement, this research used the Spanish scale developed by Benevides-Pereira et al. [51] of the UWES (Utrecht Work Engagement Scale) from the original version of Schaufeli and Bakker [52], which contains the three dimensions (three items each of them) that are part of this variable (absorption, i.e., “I am immersed in my job”; dedication, i.e., “my job inspires me”; and vigor, i.e., “at my work, I feel bursting with energy”). These dimensions were evaluated on a Likert scale ranging from 1 (low level of work engagement) to 7 (high work engagement). The validity and reliability of UWES is demonstrated by Benevides-Pereira et al. [51] in diverse environments, achieving for each of the three subscales, the following average Cronbach’s alpha: 0.88 for vigor, 0.91 for dedication, and 0.78 for absorption.

Spirituality at work was measured through the Spanish translation (using a standard back-translation procedure; the back translation matched the original items) of the scale developed by Milliman et al. [33], which is a reduced version of the Ashmos and Duchon [34] questionnaire. This scale assesses the three dimensions that make up this concept (alignment of values, eight items, i.e., “I feel positive about the values of the organization”; meaningful work, 6 items, i.e., “I experience joy in my work”; and sense of community, 7 items, i.e., “I feel part of a community in my immediate workplace”) using a Likert scale of 1 (low perception by the worker of the level of spirituality at work) to 7 (high spirituality at work). Strong reliability was demonstrated by Milliman et al. [33] with coefficient alphas ranging from 0.82 for meaningful work; 0.91 for sense of community; to 0.94.for alignment of values.

Van den Bosch and Taris [46] assessed authenticity at work through the Individual Authenticity Measure at Work (IAM), which is an adaptation of the questionnaire of authenticity developed by Wood et al. [43]. In this research, the Spanish translation of the IAM was employed: a standard back-translation procedure and the back translation matched the original items. This scale includes the dimensions presented in the theoretical framework: authentic living, i.e., “I am true to myself at work in most situations”; accepting external influence, i.e., “at work, I feel the need to do what others expect me to do”; and self-alienation, i.e., “I don’t feel who I truly am at work”. The four items that correspond to authentic living were measured on a Likert scale ranging from 1 (low level of authenticity) to 7 (high level of authenticity). The eight items (four items each) that evaluate accepting external influence and self-alienation were recoded to be consistent with the subscale for authentic living. Van den Bosch and Taris [46] demonstrated the scale’s reliability: 0.81 for authentic living, 0.83 for self-alienation, and 0.67 for accepting external influence.

### 3.3. Data Analysis

To achieve the objectives set out in this research, PLS methodology, a model of structural equations based on variance (SEM: structural equation modeling) was used. This technique was selected for different reasons; among the most relevant are the properties of the constructs that make up the research model, since the use of PLS is suitable for composite measurement models [53,54] and the remarkable adaptability of this technique to investigations carried out in social sciences research [55].

PLS evaluates both the reliability and validity of measurement models, as well as estimates the relationships between the constructs of the structural model [56]. The software used was SmartPLS 3.2.8, following a two-stage approach, since the research model includes multidimensional constructs [57]. In this research, the first-order factors are the dimensions, which become the observed indicators of the second-order constructs [58], which in this case are the variables servant leadership, spirituality at work, authenticity, and work engagement. A construct can be estimated in Mode A, i.e., reflective, or Mode B, formative. Hair et al. [59] explain that if indicators are highly correlated and interchangeable, they are reflective, and if the indicators are those that cause the latent variable and are not highly correlated (positive, negative, or even no correlated) and not interchangeable, they are formative. After reviewing the literature above, and according to the reliability analysis performed by the authors [5,33,46,51], the constructs of this research are estimated as following: servant leadership, spirituality at work and work engagement are estimated in Mode A; authenticity was estimated in Mode B. Bivariate correlations revealed in Table 1 support the suggested modes. As recommended by Ringle et al. [60], this research evaluated both measurement models and the structural model.

## 4. Results

### 4.1. Descriptive Statistics

Table 1 presents the main descriptive statistics of the first-order latent variables (dimensions of second-order constructs): the mean, standard deviation, and bivariate correlations. As seen in Table 1, the population studied mostly presents high or medium-high values in all the variables analyzed.

### 4.2. Common Method Bias (CMB)

To detect a CMB situation, a complete multicollinearity test was performed based on the variance inflation factors (VIFs) of the structural model. Table 2 presents the internal VIFs of the second-order constructs. The structural model obtained is CMB-free as its maximum VIF is 1,641, i.e., less than 3.3, a value that would indicate pathological collinearity [61].

### 4.3. PLS Models

PLS models are valued in two stages: the first seeks to verify the reliability and validity of the measurement model; the second tests the significance of the paths in the structural model.

#### 4.3.1. Measurement Model

The first- and second-order measurement models exhibit valid and reliable results. The first-order model is not presented due to its length (contact the authors of the article if required). The second-order model is shown in Table 3. The constructs of servant leadership, spirituality at work, and work engagement are estimated in Mode A. All the dimensions of these constructs satisfy the requirement of individual reliability of the elements since their loadings exceed 0.707 [62]. Additionally, they meet the reliability requirements of the construct since the Cronbach’s alpha, Jöreskog’s rho (rho_A), and composite reliability (CR) are higher than 0.7 [63]. Finally, they achieve convergent validity, with an AVE greater than 0.5 [64] and discriminative validity (Table 4), following both the criterion of Fornell and Lacker [65], which proposes comparing the square root of the AVE with the correlations between the constructs, as well as the HTMT criterion (heterotrait-monotrait), since all the values are below the 0.85 threshold [66]. The second-order construct of authenticity is estimated in Mode B. Therefore, the analyses begin by checking the potential multicollinearity between the items [55]. In this way, Petter et al. [67] indicate that a VIF above 3.3 reveals high multicollinearity. However, Ringle et al. [60] argue that multicollinearity is a concern only if VIF values exceed the critical level of 5. In this construct of authenticity, the maximum value of the VIFs is 1.532 (Table 3), so multicollinearity is not a concern. Finally, the magnitude and significance of the weights are tested, which provide information on how each dimension contributes to the construct [68]. A significance level lower or equal to 0.05 suggests that a component is relevant to the formation of the construct [55]. Authenticity dimension presents a *p*-value below 0.001 in all the weights (Table 3).

#### 4.3.2. Structural Model

Table 5 exhibits the main parameters obtained from the structural model, which enable the assessment of the statistical significance of the relationships established as hypotheses. To do this, we apply a bootstrapping technique (5000 re-samples), generating standard errors, t-statistics, *p*-values and 95% bias-corrected confidence intervals (BCCIs). The coefficient of determination (R^2^) is the main criterion for measuring the explained variance of the constructs. The results show that the structural model achieves acceptable predictive relevance for the endogenous construct work engagement as the coefficient of determination R^2^ = 0.507. However, for the variables spirituality at work and authenticity, the values obtained are R^2^ = 0.260 and R^2^ = 0.095, which is because they are constructs that help explain the variable work engagement and, in part, they are explained by servant leadership, but most of their variances are not explained by the latter.

The results obtained for the structural model confirm the positive and significant direct relationships of H2a, H2b, H3a, and H3b, as well as the individual indirect relationships relating to H2, H3, and the total indirect relationship in H4, rejecting, however, H1. This research conducted a mediation analysis in a single model at once, as in PLS is not necessary a step-wise approach [69]. Hence, as suggested by Hair et al. [59] and Nitzl et al. [69], first, the significance of the indirect effect, and second, the type of effect or mediation, were determined. In this research, the indirect positive and significant relationship of H2, H3, and the total indirect relationship in H4 have been proved, while H1 has been rejected. This means that there is a total mediation by spirituality at work and authenticity in the relationship between servant leadership and work engagement [59,69].

#### 4.3.3. Predictive Validity Assessment

Explanation and prediction are two distinct purposes that could be joined in a research study [70]. This article finds support for the predictive validity of the model presented through cross-validation with holdout samples [71], using the PLS prediction algorithm [72] available in SmartPLS software version 3.2.8 [60]. This method, as suggested by Shmueli et al. [72], uses holdout samples to generate and evaluate these predictions, splitting the full database (*n* = 270) randomly into *k* equally sized subsets of data (*k* = 10; i.e., 10-folds). Then, the algorithm predicts each fold (holdout sample) with the remaining *k*-1 subsamples, which become the training sample. The positive values of Q² imply that the prediction error of PLS results is smaller than the prediction error of only using the mean values. Therefore, the proposed research model provides appropriate predictive ability for work engagement, spirituality at work, and authenticity constructs and for the dimensions that compose them (see Table 6).

## 5. Discussions

Religious organizations are key actors in today’s society and global economy, representing a considerable part of the service sector in areas such as social services, education, and health. Particularly, faith-based institutions in the social sector play an important role not only in economic terms but also in the spiritual realm, as they have certain unique characteristics that distinguish them from other organizations. Their main objective and mission are to transmit their identity values through the provision of an essential service. Therefore, their workers are critical to fulfilling their mission of transmitting their values, as well as to achieving long-term sustainability and viability, since the quality of the services provided will depend on the workers, differentiating one organization from others. In the context of less religious people and greater collaboration of the laity, the quality of an organization’s services will increase with the engagement of its workers [1]. In this context, the leadership perceived by workers in their superiors represents a critical piece, as well as the spirituality and authenticity they experience, as these are sources of positive effects on employees, such as work engagement. Researchers argue that work engagement is essential to organizational success [73,74].

The objective of this research is to deepen the study of workers in religious organizations in the social sector, and, in particular, the link between workers’ perceived level of servant leadership in their superiors and work engagement, as well as the role that authenticity and spirituality at work play in this relationship. To this end, we analyzed a Spanish Catholic organization in the social sector, obtaining 270 valid surveys from its employees. First, Hypothesis 1 of this article proposes that a higher perception of servant leadership style in superiors has a significant positive effect on the work engagement of employees in social religious organizations [12,13,23]. The results do not support the behavioral theories explained in the theoretical framework, that defend that servant leadership may influence performance [10] and generate employee’s beneficial behaviors for the organization [28], as the structural model did not show a significant relationship between servant leadership and worker´s engagement. However, it is noteworthy that although this hypothesis is supported by previous literature, the results of this study do not support it in the target organization. These results are in line with other research that found no positive relationship between a servant leadership style perceived by employees and greater work engagement, such as a study conducted among engineering consultants at an international U.S. firm [31]. However, the study’s author explains that although this relationship is not significant, in comparing the level of employee engagement per department with its percentage of servant leaders, the departments with more servant leaders seem to have higher engagement. Here arises a very controversial issue as these results suggest that, while in other types of organizations, a style of servant leadership stimulates this positive energy in workers, religious organizations in the social sector need to complete this strategy with another set of tools or stimuli, such as fomenting authentic and spiritual attitudes.

Second, this study hypothesizes that spirituality at work plays a mediating role in the previous relationship. In other words, a higher perception of servant leadership entails greater spirituality at work [36,37], which in turn generates greater work engagement [33]. The results confirm that these relationships are significant, which favors a strategy of promoting spirituality at work so that, in this way, religious organizations in the social sector that promote servant leadership reinforce the work engagement of their employees. This is possible because workers in such organizations often feel identified with these entities’ mission and values, and promotion of spirituality at work would allow them to find their life purpose at work, feel a strong connection with other members of the organization and perceive an alignment of their values and beliefs with those of the organization. These results bring new contributions to behavioral servant leadership theories, which, to our knowledge, have not studied if servant leaders generate an attitude of spirituality at work in their followers. Moreover, these results are consistent with the target organization, which develops different activities that promote spirituality at work, such as periodically training activities and courses oriented towards the identity and mission of the institution.

Third, the results confirm that authenticity plays a mediating role in the relationship between servant leadership and work engagement. These data are in line with studies such as Ramsey [14] that show that servant leadership is positively related to authenticity, as well as that the last one has positive effects on work engagement [1,49,50]. This is possible because servant leaders are both authentic and ethical, increasing the number of followers through the unique characteristics of this leadership [75], which connects emotionally with followers by promoting employee engagement [76,77]. Hence, these results are complementing the behavioral theories of servant leadership, as to our knowledge, at the moment they do not demonstrate that perception of servant leadership may influence on follower´s authenticity. Probably, the personality and personal values of the employees working in this type of institutions have determined the obtained result, as they are usually individuals that appreciate entities where they can act by their ideas and beliefs [78]. Additionally, the obtained results are in line with the target organization, as it promotes professionals motivated and identified with its mission and values, that help them to develop their social intervention projects adapted to the needs of the beneficiaries, or in other words, the analyzed institution promotes engaged workers with authentic attitudes. In today’s context, where authenticity is very appreciated by most of the employees, finding ways to promote it is of first importance and merits serious attention from researchers, which in turn fosters their work engagement.

Finally, this study also proves that the most significant effect of servant leadership on work engagement occurs through the total indirect effect. That is, a perceived servant leadership strategy does not by itself produce an increase in employees’ work engagement; however, when social religious organizations that practice this style of leadership foster a working climate promoting attitudes of spirituality at work and authenticity together, worker engagement is strengthened. In other words, spirituality and authenticity acting together exert a total mediation effect, which is more significant than the effects of their individual action. This finding highlights the importance of these variables in the little-explored context of religious organizations, where workers find a way to make sense of their lives through their work. These variables are goals for this type of organization since their workers find in them and their work activities several personal and spiritual incentives different from the economic ones. Then, these conclusions convert spirituality and authenticity at work into two instruments of the organization to help to increase the engagement of those workers who perceive a servant leadership style. Moreover, this study also gives the opportunity to improve the quality of life of employees, as it promotes attitudes of authenticity and spirituality at work, which are very demanding qualities in the labor market nowadays [19,20,48].

Hence, the main contribution of this research lies in demonstrating that, in a context of perceived servant leadership, the probability of being more engaged in the organization could increase among those members who can live their spirituality and act authentically. These obtained conclusions are probably a consequence of the peculiarities of the target context. First, it is a social organization. Its main aim is to help people in a situation of risk or social exclusion, attending their real needs. In many cases, these social jobs are personally demanding, and hence, vocational positions [18]. This makes that people working there probably share the values of the organization, as they prefer the benefits of working in an activity that they feel rewarding and being able to act authentic and live their spirituality at work, that other kind of remuneration they could get in a different company. Second, it is a religious institution, which implies its objective is to transmit the predominant values in their mission while providing its services. The target organization has a common culture that is managed in a centralized way, to guarantee the unity of values and monitoring of the mission. Moreover, it looks for professionals that are motivated and identified with their mission and values in their selection process. This set of circumstances shows that promoting attitudes of spirituality and authenticity at work is an objective for this institution and becomes an instrument to achieve work engagement.

These results contribute to the corporate governance of religious institutions in two ways. First, to identify what types of attitudes should be promoted in the organizational context with actual employees (such as implementing training activities or training courses that encourage spirituality and authenticity levels), or even attitudes that could be sought after when selecting potential employees in the human resources selection process. Second, to identify what kind of values should be promoted among employees and look for in those in the selection process. Looking for potential workers that share the values of the organization is going to favor the institution´s mission. This is because those workers who share the organization´s values are going to be able to feel more authentic and spiritual, and hence, more engaged in a context of perceived servant leadership. Therefore, the values that lead the organizational culture are going to be transmitted by their workers while developing their activity.

## 6. Conclusions

The results of this research are important to better understanding employees’ view among religious entities in the social sector. Although there are studies noting the importance of perceiving a servant leadership strategy among followers, to our knowledge, none focus on demonstrating the fundamental roles of authenticity and spirituality at work in achieving a greater work engagement in followers through this leadership strategy, neither in these types of organizations. This research shows two fundamentals conclusion. First, although in others types of entities, servant leadership generates work engagement, among employees in religious organizations of the social sector, perceived servant leadership does not give rise to such engagement by itself among followers. Second, in a context like the target organization, where servant leadership is perceived, the engagement of these workers comes through two mediating variables: the possibility of being authentic and living one’s spirituality at work. Understanding the perspective of employees is critical for managers of these organizations to obtain the greatest possible benefit when they implement a style of leadership based on service to others. In this line, this research helps to manage the delicate balance between effectiveness, efficiency, mission, and vision that drive social religious organizations. These findings are also key to the corporate governance of these religious entities and their leaders, because even if they work hard to promote that their employees perceive a servant leadership environment, if they do not also encourage workers to act according to their values and beliefs and freely live their spirituality at work with all that implies, they may not achieve an increase in their engagement

Hence, this research makes some fundamentals contributions to behavioral theories of servant leadership. On the one hand, it contributes to social learning theory in explaining that those servant leaders, through how their behavior is perceived by their followers, may stimulate attitudes of spirituality and authenticity at work in their followers, which probably will affect their work engagement. On the other hand, this study also provides some valuable insights into the social identity theory. Servant leaders make employees feel part of the organization through their follower center nature. When employees self-identify with the group, they are more likely to develop their spirituality and authenticity at work, which finally, would enhance their engagement.

## 7. Limitations and Future Lines of Research

This research makes significant contributions but also exhibits some methodological limitations that should be noted. First, although the results could be replicated in other religious entities in the social sector, this research is carried out in a Catholic organization located in the south of Spain. Future lines of research could develop this study in other faith-based organizations in the social sector outside and in other locations of Spain. We would like to emphasize that although this research takes place in a social religious institution, the results obtained could be valuable for entities in other sectors and for-profit companies that wish to base their management models on values. These management strategies are increasingly demanded by both, companies which seek new leadership styles that go beyond economic incentives and make employees to find meaning in their work, achieving a more engaged and committed workforce, and workers who demand entities that enable them to act according to their values and beliefs. Hence, future investigations should confirm that this study is also valid within the framework of for-profit entities. Second, the information in this study was obtained through self-administered questionnaires, which could cause a response bias; according to De Carvalho et al. [41], this could be addressed by supplementing such questionnaires with other more objective measures. Third, due to the cross-sectional nature of the data, although theoretical arguments contribute to cause-and-effect relationships, a longitudinal study would help to address the potential existence of causal relationships between variables.

This research offers a wide range of future lines of investigation. Although the study has been performed for the whole organization, as it is a unique organization that shares the same leaders, mission, and values, it may influence that at the moment of the study the employees were working at different social intervention projects or social centers. Hence, future research could perform a multilevel analysis, comparing the obtained general results by territorial areas or social intervention projects. In addition, we utilized the follower´s vision questionnaire, as we believe that their work engagement, spirituality, and authenticity are going to depend on how the employees perceive their supervisors. However, this study does not analyze the follower´s version. It could also be interesting future research to study the effect of the leader’s perception on the analyzed dependent variables. Finally, since this study compares the mediating effects of spirituality at work and authenticity in the relationship between servant leadership and work engagement, future research could study these mediating effects with other types of leadership, such as authentic or transactional leadership, and assess their effect on other outcome variables significant to these organizations, such as the subjective wellbeing of workers. In addition, other positive constructs could play a mediating role in the relationship between servant leadership and work engagement; variables like organization-based self-esteem could be studied [79].

## Figures and Tables

**Table 1:** Descriptive statistics and intercorrelations of the study variables.

Variable		Range	Mean	SD	1	2	3	4	5	6	7	8	9	10	11	12
1	Openness	1–5	3.96	0.99	1											
2	Stewardship	1–5	4.23	0.82	0.744 **	1										
3	Vision	1–5	4.15	0.83	0.571 **	0.716 **	1									
4	Alignmt. Values	1–7	6.16	0.85	0.427 **	0.444 **	0.321 **	1								
5	Meaningful Work	1–7	6.07	0.89	0.384 **	0.311 **	0.217 **	0.621 **	1							
6	Sense of Community	1–7	5.81	1.07	0.507 **	0.508 **	0.390 **	0.776 **	0.567 **	1						
7	Authentic living	1–7	5.92	0.66	0.197 **	0.137 *	0.076	0.187 **	0.298 **	0.240 **	1					
8	Ac. External Infl.	1–7	5.16	1.34	0.226 **	0.170 **	0.074	0.255 **	0.227 **	0.286 **	0.142 *	1				
9	Self-alienation	1–7	6.15	1.14	0.307 **	0.202 **	0.100	0.397 **	0.369 **	0.419 **	0.292 **	0.452 **	1			
10	Absorption	1–7	5.59	0.90	0.137 *	0.085	0.055	0.321 **	0.459 **	0.290 **	0.196 **	.036	0.169 **	1		
11	Dedication	1–7	6.25	0.78	0.367 **	0.272 **	0.124 *	0.492 **	0.726 **	0.470 **	0.280 **	0.299 **	0.443 **	0.510 **	1	
12	Vigor	1–7	5.81	0.85	0.343 **	0.183 **	0.094	0.458 **	0.656 **	0.443 **	0.280 **	0.322 **	0.508 **	0.466 **	0.747 **	1

** *p* < 0.01; * *p* < 0.05.

**Table 2:** Full collinearity VIFs.

	Work Engagement
Servant Leadership	1.358
Spirituality at Work	1.641
Authenticity	1.342

**Table 3:** Measurement model. Reliability and convergent validity.

Variable		Loadings	Weights	VIF	Cronbach’s Alpha	rho_A	Composite Reliability	Average Variance Extracted (AVE)
1	Servant Leadership (Reflective)				0.864	0.929	0.914	0.780
1.1	Openness (Reflective)	0.912 ***						
1.2	Stewardship (Reflective)	0.805 ***						
1.3	Vision (Reflective)	0.928 ***						
2	Spirituality at work (Reflective)				0.853	0.856	0.910	0.772
2.1	Alignment of Values (Reflective)	0.902 ***						
2.2	Meaningful Work (Reflective)	0.852 ***						
2.3	Sense of community (Reflective)	0.881 ***						
3	Authenticity (Formative)							
3.1	Authentic living (Formative)		0.333 ***	1.240				
3.2	Ac. External Influence (Formative)		0.312 ***	1.284				
3.3	Self-alienation (Formative)		0.622 ***	1.532				
4	Work engagement (Reflective)				0.836	0.871	0.900	0.752
4.1	Absorption (Reflective)	0.776 ***						
4.2	Dedication (Reflective)	0.921 ***						
4.3	Vigor (Reflective)	0.898 ***						

The loading and weights significance was estimated by bootstrap 95% confidence interval (based on n = 5000 subsamples). *** *p* ≤ 0.001 (based on t (4999), two-tailed test).

**Table 4:** Discriminant validity.

Fornell-Lacker					Heterotrait-Monotrait Ratio (HTMT)			
	A	WE	SaW	SL		WE	SaW	SL
A	*N/a*				WE			
WE	0.528	0.867			SaW	0.773		
SaW	0.501	0.672	0.879		SL	0.300	0.582	
SL	0.308	0.288	0.510	0.883				

A, authenticity; SaW, spirituality at work; SL, servant leadership; WE, work engagement.

**Table 5:** Structural model.

*R*^2^*WE = 0.507*; *R*^2^*SaW = 0.2598*; *R*^2^*A = 0.095* Relationship	Path Coefficient	*T*-Statistics	*p*-Values		2.5%	97.5%	Significance	Hypothesis
***Direct Effects***								
SL -> WE	−0.092	1.863	0.062		−0.185	0.005	No Sig.	H1
**SL -> SaW**	**0.510**	**9.664**	**0.000**	***	**0.398**	**0.606**	**Sig.**	**H2a**
**SaW -> WE**	**0.587**	**9.135**	**0.000**	***	**0.453**	**0.703**	**Sig.**	**H2b**
**SL -> A**	**0.308**	**5.006**	**0.000**	***	**0.169**	**0.416**	**Sig.**	**H3a**
**A -> WE**	**0.262**	**3.767**	**0.000**	***	**0.126**	**0.398**	**Sig.**	**H3b**
***Individual Indirect Effects***								
**SL -> SaW -> WE**	**0.299**	**7.067**	**0.000**	***	**0.223**	**0.387**	**Sig.**	**H2**
**SL -> A -> WE**	**0.081**	**2.923**	**0.003**	**	**0.036**	**0.145**	**Sig.**	**H3**
***Total Indirect Effect***								
**SL -> WE**	**0.380**	**8.217**	**0.000**	***	**0.287**	**0.466**	**Sig.**	**H4**

Bootstrapping 95% confidence intervals bias corrected (based on n = 5000 subsamples). *** *p* ≤ 0.001; ** *p* ≤ 0.01; * *p* ≤ 0.05 (based on t(4999), two-tailed test). Relevant relationships in bold. A, authenticity; SaW, spirituality at work; SL, servant leadership; WE, work engagement.

**Table 6:** Partial least squares prediction assessment.

Construct Prediction Summary	
	**Q^2^**
Spirituality at Work	0.246
Authenticity	0.079
Work Engagement	0.075
**Dimension Prediction Summary**	
	**Q^2^**
Alignment of Values	0.193
Meaningful Work	0.108
Sense of Community	0.272
Authentic living	0.030
Ac. External Infl.	0.038
Self-alienation	0.067
Absorption	0.012
Dedication	0.089
Vigor	0.060
